# The unfolded protein response modulators GSK2606414 and KIRA6 are potent KIT inhibitors

**DOI:** 10.1038/s41419-019-1523-3

**Published:** 2019-04-01

**Authors:** Mohamed Mahameed, Thomas Wilhelm, Odai Darawshi, Akram Obiedat, Weiss-Sadan Tommy, Chetan Chintha, Thomas Schubert, Afshin Samali, Eric Chevet, Leif A. Eriksson, Michael Huber, Boaz Tirosh

**Affiliations:** 1 Institute for Drug Research, The Hebrew University of Jerusalem, Jerusalem, Israel; 2 Institute of Biochemistry and Molecular Immunology, Medical School, RWTH Aachen University, Aachen, Germany; 3 Apoptosis Research Centre, National University of Ireland Galway, Galway, H91 TK33 Ireland; 4 2bind GmbH, Am BioPark 11, 93053 Regensburg, Germany; 5 INSERM U1242, Université de Rennes, Rennes, France; 6 Centre de Lutte Contre le Cancer Eugène Marquis, Rennes, France; 7 Department of Chemistry and Molecular Biology, University of Gothenburg, Göthenburg, Sweden

## Abstract

IRE1, PERK, and ATF6 are the three transducers of the mammalian canonical unfolded protein response (UPR). GSK2606414 is a potent inhibitor of PERK, while KIRA6 inhibits the kinase activity of IRE1. Both molecules are frequently used to probe the biological roles of the UPR in mammalian cells. In a direct binding assay, GSK2606414 bound to the cytoplasmic domain of KIT with dissociation constants (*K*_*d*_) value of 664 ± 294 nM whereas KIRA6 showed a *K*_*d*_ value of 10.8 ± 2.9 µM. In silico docking studies confirmed a compact interaction of GSK2606414 and KIRA6 with KIT ATP binding pocket. In cultured cells, GSK2606414 inhibited KIT tyrosine kinase activity at nanomolar concentrations and in a PERK-independent manner. Moreover, in contrast to other KIT inhibitors, GSK2606414 enhanced KIT endocytosis and its lysosomal degradation. Although KIRA6 also inhibited KIT at nanomolar concentrations, it did not prompt KIT degradation, and rescued KIT from GSK2606414-mediated degradation. Consistent with KIT inhibition, nanomolar concentrations of GSK2606414 and KIRA6 were sufficient to induce cell death in a KIT signaling-dependent mast cell leukemia cell line. Our data show for the first time that KIT is a shared target for two seemingly unrelated UPR inhibitors at concentrations that overlap with PERK and IRE1 inhibition. Furthermore, these data underscore discrepancies between in vitro binding measurements of kinase inhibitors and inhibition of the tyrosine kinase receptors in living cells.

## Introduction

The endoplasmic reticulum (ER) is the entry into the secretory pathway, where proteins destined for secretion or membrane embedding undergo folding and where multi-subunit complexes are assembled. ER functionality requires the constant adjustment of its folding capacity to the protein folding demand. Thus, when perturbations in homeostasis occur owing to multiple reasons, such as viral infection, differentiation, or alterations in growth conditions, collectively referred to as ER stress, eukaryotic cells activate an adaptive signaling pathway called the unfolded protein response (UPR)^1^.

The mammalian UPR is operated by three canonical arms termed on their proximal ER stress sensors: IRE1, PERK, and ATF6. The first two are serine/threonine kinases that are activated by auto-transphosphorylation in response to ER stress. IRE1 is also an endoribonuclease (RNase), controlled by its phosphorylation and oligomerization state^2^. IRE1 RNase impinges on cell fate in a manner that is proportional to the magnitude of ER stress. If ER stress is moderate, IRE1 primarily through the non-canonical splicing of XBP1 mRNA improves the removal of unfolded proteins and restores ER homeostasis. However, if stress is irremediable, IRE1 promotes cell death, by RNA degradation of various RNA (RIDD)^3^. PERK, which is activated similarly to IRE1, is an eIF2α kinase. The phosphorylation of eIF2α attenuates global protein translation and, however, leads to the preferred translation of selective mRNAs, such as the one encoding transcription factor ATF4^4^.

Cellular and animal models using gain and loss of function of various UPR proteins have shown a potential involvement of the UPR in major pathologies, such as diabetes, neurodegeneration, and cancer. This has promoted the development of drugs that probe different elements of the UPR signaling, hoping to identify potential disease modulators. The development of PERK inhibitors was primarily motivated by genetic evidences that implicate PERK as contributing to cancer initiation, progression, and facilitation of the resistance of cancer to chemotherapy. GSK2606414 (termed hereafter as GSK414) has been identified as a selective PERK inhibitor following optimization of a lead molecule identified from a kinase inhibitors library. GSK414 is highly potent for PERK with an in vitro IC_50_ of lower than 1 nM. Despite the sub-nanomolar IC_50_ of GSK414, 30 nM were needed to completely block PERK autophosphorylation under conditions of extreme ER stress^5^. While having promise as an anti-cancer agent, animal studies showed the development of hyperglycemia and reduction of serum insulin upon long-term treatment, effects consistent with the importance of PERK for insulin secretion^6^. Because GSK414 is directed, as all kinase inhibitors, to the ATP binding site of PERK, a concern was raised regarding its selectivity to PERK. According to the original report on GSK414 characterization, in a panel of 294 kinases the most sensitive kinase after PERK was the tyrosine kinase receptor KIT with IC_50_ of 154 nM^5^. Recently, GSK414 was also demonstrated to inhibit RIPK1, a kinase involved in TNFα-mediated cell death. The IC_50_ of GSK414 for RIPK1 was similar to that of PERK in living cells^7^. The kinase activity of IRE1 was shown to allosterically regulate its RNase activity^8^. Accordingly, inhibitors of IRE1 kinase activity were suggested to have an advantage over blockers of its nuclease activity, which exert their function by an exposed aldehyde that limits drug stability and leads to off-target activities^9^. Developed originally from APY29, a molecule that activated IRE1 RNase activity, KIRA6 was shown to bind the ATP binding site of IRE1, to repress its oligomerization and thereby its RNase activity. Accordingly, KIRA6 at 100–500 nM concentrations rescued β islet cells from tunicamycin-induced ER stress toxicity^8^. Thus, KIRA6 was proposed as a potential drug for certain types of diabetes. No off-targets were identified so far for KIRA6.

KIT (also known as CD117 or c-Kit) is a type III receptor tyrosine kinase (RTK), predominantly expressed in germ cells, hematopoietic progenitor cells, mast cells, intestinal epithelium, melanocytes, breast ductal epithelium, neurons, and the pacemaker cells of the gut. KIT plays a crucial role in growth and development, cell survival, metabolism, and differentiation. Upon binding to its ligand, stem cell factor (SCF), KIT activates multiple downstream signal transduction pathways including RAS/ERK, PI3K/AKT, phospholipase C, JAK/STAT, and Src kinase pathways^10^. In certain types of cancers, such as gastrointestinal stromal tumors or mastocytosis, KIT is the major target for therapy, primarily by imatinib.

Owing to the fact that GSK414 has been demonstrated to have off-target kinase inhibitory effects and the fact that KIT was proposed to be a potential target for GSK414, we investigated this in detail. We show that GSK414 inhibits KIT signaling at sub-micromolar concentrations, which are typically used to inhibit PERK. Unexpectedly, KIRA6 also inhibited KIT at a similar concentration range. Direct binding measurements revealed that GSK414 associated more tightly than KIRA6 to the KIT cytoplasmic domain. GSK414 not only blocked KIT signaling, but also directed the kinase for lysosomal degradation, an unusual effect of an antagonist. KIRA6, on the other hand, probably through competitive binding protected KIT from GSK414-mediated degradation. Both inhibitors compromised the viability of cells that rely on KIT signaling for survival. Our data highlight KIT as a shared target of seemingly unrelated UPR modulators and demonstrate a pharmacological way to facilitate KIT degradation by a kinase inhibitor.

## Experimental procedures

### Cell lines and culturing conditions

MEL526, HepG2, and 293T cells were cultured in high glucose Dulbecco’s Modified Eagle medium (DMEM high glucose, Sigma-Aldrich) supplemented with 10% fetal bovine serum (FBS, Invitrogen), 2 mM L-glutamine (Biological Industries, Israel), 1% penicillin–streptomycin solution (Biological Industries, Israel), and 1 mM sodium pyruvate (Biological Industries, Israel). HMC-1.1 cells were cultured in Iscove’s Modified Dulbecco’s Medium (IMDM, ThermoFisher Scientific) supplemented with 10% Calf serum-Iron Fortified (Sigma-Aldrich) 2 mM L-glutamine, 1% penicillin–streptomycin solution, and 1.2 mM monothioglycerol (Sigma-Aldrich). Cells were incubated at 37 °C under 5% CO_2_.

### Chemical reagents

GSK2606414 (TOCRIS #5107), AMG44 (TOCRIS #5517), Necrostatin-1 (Apollo Scientific #BIM0213), Dasatinib (Sigma-Aldrich#CDS023389), KIRA6 (Calbiochem#532281), Cycloheximide (Sigma-Aldrich #66819), Bortezomib (Calbiochem #504314), Bafilomycin A1 (Sigma-Aldrich #B1793), human SCF (Peprotech #CYT-255).

### Measurement of binding constant to recombinant cytoplasmic portion of KIT by MicroScale Thermophoresis (MST)

Proteins used in the assay, KIT wild type (ProQinase #0997-0000-1) and D816V (ProQinase #0946-0000-1) were labeled with RED-tris-NTA fluorescent dye according to the instructions in the user manual (RED-tris-NTA 2nd generation, NanoTemper # MO-L018). 100 µl of 200 nM protein solution is mixed with 100 µl of 100 nM dye solution and incubated for 30 min at RT in the dark. MicroScale Thermophoresis (MST) assay was performed with labeled protein in standard Monolith NT.115 capillaries (NanoTemper #MO-K022) in an MST buffer (50 mM Tris–HCl pH 7.8, 150 mM NaCl, 10 mM MgCl_2_, 0.05% Tween-20). The compounds were serially diluted 1:1 with MST buffer reducing the compound concentration by 50% in each of the 16 dilution steps. For the direct binding measurement, 5 µl of labeled protein at a final concentration of 100 nM was mixed with 5 µl of the respective compound dilution and incubated in capillaries for 30 min at RT. For GSK414 and KIRA6 a final DMSO concentration of 5% was used, for imatinib 2% methanol was used in each capillary. All measurements were performed at 60% MST power and 40% excitation power using the Monolith NT.115 instrument (NanoTemper Technologies). Data analysis of three independent experiments was performed using NanoTemper analysis software (MO.Affinity Analysis v2.3), whereas the hot values (laser on) were set to 2 respectively 5 s. The presented MST data meet the relevant qualitative criteria, such as signal to noise ratio >7, absence of aggregation effects, and absence of unspecific adsorption to glass capillaries. Merged data of three independent experiments are presented as fraction bound normalized (0 = unbound, 1 = bound state).

### Generation of knock-out cells using CRISPR/Cas9

gRNA was designed according to Doench et al.^11^ gRNA was cloned into PX459 vector according to the Zhang lab protocols^12^. MEL526 cells were transfected using Mirus2020 (3 µl per 1 µg DNA) and then underwent puromycin selection (2 µg/ml) for 48 h followed by single-cell cloning by limiting dilution. Clones were screened by immunoblotting for the relevant protein. Two gRNA sequences were simultaneously used for human PERK: 5′-CCGAGGCTCCTGCTCTCCCG-3′; 5′-GAATATACCGAAGTTCAAAG-3′, gRNA sequences for human IRE1: 5′-CTTTTATGTCTGGCAGCGGG-3′.

### Western blotting

Cells were either trypsinized or directly harvested by cell scraping, centrifuged at 1000 rcf for 5 min, and washed twice in cold PBS. For cell lysis, RIPA buffer supplemented with protease and phosphatase inhibitors was added in a volume of about four times the cells’ pellet, then vibrated for 20 min at 4 °C. Lysates were cleared by centrifugation at 20,000 rcf for 30 min at 4 °C. 5× reduced Laemmli sample buffer was added, boiled for 5 min at 95 °C, and loaded on SDS-PAGE. The same procedure was performed for non-reducing SDS-PAGE using 5× non-reduced Laemmli (lacking DTT). Following SDS-PAGE, gels were blotted onto PVDF membranes using BioRad PowerPac^TM^. Blots were blocked in 10% skim milk in TBST buffer for 1 h at room temperature. The following primary antibodies were used: rabbit anti-phospho KIT (Tyr719) (Cell Signaling #3391); rabbit anti-KIT antibody (Cell Signaling #3074); rabbit anti-PERK (Cell Signaling #5683); rabbit anti-IRE1 antibody (Cell Signaling #3294); rabbit anti-EGFR antibody (Cell Signaling #4267), mouse anti-MET antibody (Cell Signaling #3127), rabbit anti-GAPDH antibody (Cell Signaling #2118); rabbit anti-phospho-Stat5 antibody (Cell Signaling #9351); rabbit anti-phospho-Erk1/2 (Thr202/Tyr204) (Cell Signaling #4370); rabbit anti-α/β tubulin (Cell Signaling, #2148), mouse anti-puromycin antibody, clone 12D10 (Millipore #MABE343); polyclonal rabbit anti-p97 was provided by Dr. Hidde Ploegh (Boston Children’s Hospital, Boston, MA). Secondary HRP-conjugated goat anti-rabbit and anti-mouse (Jackson Immunoresearch, West Grove, PA) were used. Blots were developed in Bio-Rad ChemiDoc™ XR. Quantification was done by densitometry by using Image Lab™ software.

### Flow cytometry

Cells were harvested, centrifuged, and washed twice in PBS and resuspended in 100 µl of PBS. Fluorophore-conjugated antibody was added according to the manufacturer’s instructions, followed by filtration through a 100 µM strainer directly to FACS tubes. Analysis was performed using Cytoflex FACS using the CytExpert software for data processing. Conjugated antibodies used: APC-anti-human CD117 (KIT) APC (Biogems #19211-80); PE-anti-human CD221 PE (IGF-1R) (Miltenyi Biotech #130-103-939); FITC-anti-human CD71 (transferrin receptor) (BioLegend #334013), APC-anti-human-c-MET (Sino Biological #10692-R271-A); Alexa 488-anti-human EGFR (BioLegend #352907).

### qPCR analyses

Total RNA was isolated using TRI-reagent (Bio-Lab). cDNA was synthesized using 1 µg RNA with qScript^™^ cDNA Synthesis KIT (Quanta Biosciences) according to the manufacturer’s instructions. Bio-Rad iTaq™ universal SYBR^®^ Green Supermix was used for quantitative Real-Time PCR analyses. hRPLP0 was used as an endogenous housekeeping gene for qPCR quantification. Analysis was performed on CFX Connect™ Real-Time PCR Detection System (Bio-Rad) using the Bio-Rad CFX manager 3.1 software. The following primers were used: For human KIT: forward: 5′-CGTTCTGCTCCTACTGCTTCG-3′, reverse: 5′-CCCACGCGGACTATTAAGTCT-3′; for RPLP0: forward 5′-CCAACTACTTCCTTAAGATCATCCAACT-3′, reverse: 5′-ACATGCGGATCTGCTGCA-3′.

### Protein stability assay

Protein stability experiments were performed using cycloheximide chase. Cells were harvested in 15 ml tubes, washed twice with cold PBS, and re-suspended again in PBS. Cycloheximide (50 μg/ml) was added to each tube and immediately incubated in 37 °C water bath during the whole experiment. At each time point, cells were homogenously re-suspended by pipetting and the same volume was taken. Cells were centrifuged and the cell pellets were kept at −80 °C till the end of the chase. Samples were processed for Western blotting as detailed above.

### Immunofluorescence and microscopy

MEL526 cells were plated in an 8-well chamber (2 × 10^4^ cells per well). A day after, the medium was replaced with fresh medium containing either DMSO or GSK414 (1 μM) with bafilomycin A1 (100 nM) and then incubated for 12 h. Cells were washed three times with cold PBS and fixated by adding 400 μl of 4% paraformaldehyde (diluted in PBS) for 10 min at room temperature. After washing, cells were permeabilized with 0.25% Triton X-100 for 10 min followed by BSA (2% in PBS) blocking for 30 min. Rabbit anti-KIT antibody was added (1:250) and incubated overnight at 4 °C. Cells were washed and goat anti-rabbit-Alexa Fluor 647 was added (1:500) for 1 h in room temperature. 200 μl DAPI (300 nM) was added for 10 min followed by PBS washings. Images were taken by inverted microscope IX83 (Olympus) equipped with oil immersion UPLSAPO ×100 1.4NA objective lens. All images were subsequently processed by Fiji V1.51W. Images were processed using the unsharp mask with radius set to 2 and mask weight 0.6 and brightness and contrast were adjusted equally to all images.

### Biotinylation of cell surface proteins

Biotin labeling of cell surface proteins was performed using Pierce Cell Surface Protein Isolation KIT (ThermoFisher #89881).

### Propidium iodide staining and XTT viability assay

To evaluate the dead cell percentage using flow cytometry analysis, 1 µl of propidium iodide (1 mg/ml) was added to the cells before the analysis. The same number of events were recorded and PE positive cells were gated and considered as dead cells.

Metabolic activity was measured using the XTT Cell Proliferation Kit II (XTT) (Roche). Cells were seeded in microplates at a density of 3.5 × 10^5^ cells/ml (suspension culture grade, 96 wells, flat bottom) in a final volume of 100 μl culture medium per well in a humidified atmosphere (37 °C, 5% CO_2_) for 72 h. After the incubation period, 50 μl of the XTT labeling mixture was added to each well (final XTT concentration 0.3 mg/ml). Incubation of the microplate was for 3–4 h in a humidified atmosphere (e.g., 37 °C, 5% CO_2_).

Spectrophotometrical absorbance of the samples was measured using a microplate reader. The wavelength used to measure the absorbance of the formazan product of the XTT assay was 475 nm and the reference wavelength was 650 nm. Sample values at 475 nm were subtracted with medium controls (blanked) resulting in delta blanked values. Total absorbance was calculated by subtraction of delta blanked values (475 nm) with their reference values at 650 nm. These absorbance values (A475 nm–A650 nm) are shown in Fig. 6b.

### In silico docking

The 1.90 Å resolution human KIT crystal structure with inhibitor PLX647 bound^13^ was chosen for the docking simulations due to the high resolution, the complete chains, and the similarity of the co-crystallized ligand PLX647 to GSK2606414 and KIRA6. The protein was prepared using the automated script QuickPrep in MOE 2018.01. This includes adding hydrogens, setting protonation according to physiological pH and local surrounding, and structure refinement. Prior to docking, water and ligand were deleted. Partial charges based on the AMBER10:EHT force field were added to all atoms. The ligands were constructed using Builder in MOE 2018.01, partial charges based on the MMFF94x force field were added, after which the molecules were protonated according to physiological pH, and the structures minimized. Docking was performed using the Dock module in MOE 2018.01. First, the Site Finder module was used to identify the binding pocket coinciding with the co-crystallized pocket (the main site identified). Docking was performed towards the active site using the Triangle Matcher algorithm and London DG scores. From the 100 poses recorded, the 10 best scoring poses were refined using the Induced fit algorithm and GBVI/WSA DG to compute the final score.

### Statistical analysis

We applied a two-tailed Student’s *T* test (for *n* = 4) or Mann–Whitney *U* test (for *n* = 3) to determine statistical significance.

## Results

### GSK414 inhibits KIT signaling in a PERK-independent manner in living cells

**Fig. 1:**
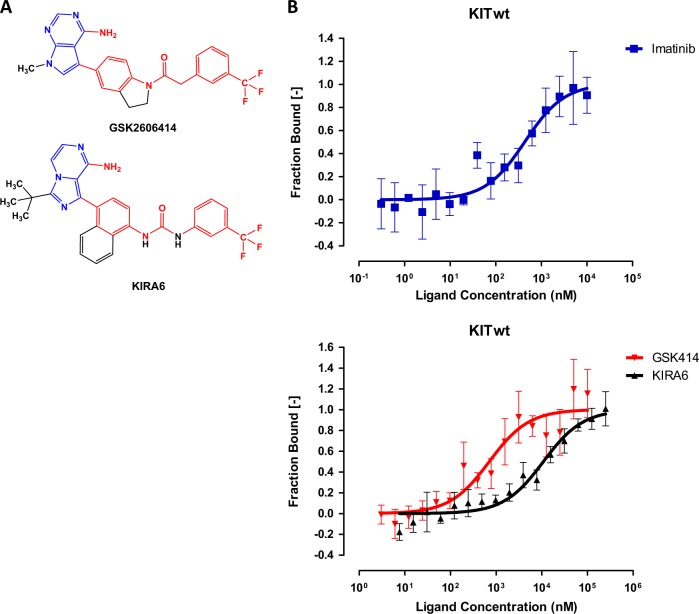
Measurement of the direct binding of GSK414 and KIRA6 to the cytoplasmic portion of KIT by MicroScale Thermophoresis (MST). **a** Chemical structures of GSK414 and KIRA6. The shared chemical elements of both compounds are shown in red color while the structurally similar parts are shown in blue color. **b** Binding curves of cKIT WT to imatinib (blue) *K*_*d*_ 383 ± 146 nM, GSK414 (red) *K*_*d*_ 664 ± 294 nM, and KIRA6 (black) *K*_*d*_ 10.8 ± 2.9 µM from three independent repeats of the MST experiment

**Fig. 2:**
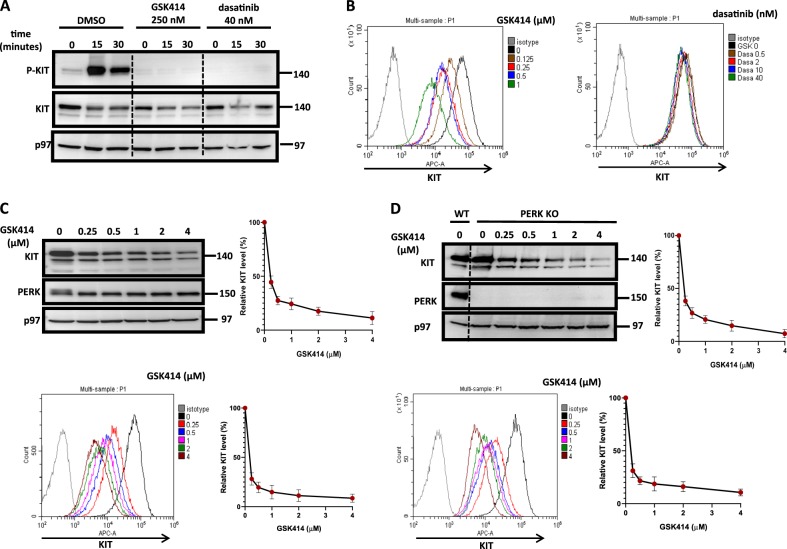
GSK414 inhibits and downregulates KIT in a PERK-independent manner. **a** Levels of P-KIT and total KIT in MEL526 WT pretreated for 1 h either with DMSO, GSK414 (250 nM), or dasatinib (40 nM) followed by SCF (50 ng/ml) addition for up to 30 min. **b** Flow cytometry analysis for surface KIT following GSK414 or dasatinib treatments for 16 h in MEL526 WT. **c** Immunoblotting (top) and flow cytometry analysis (down) for KIT in MEL526 WT following GSK414 treatments for 16 h. Western blot and surface KIT quantifications are expressed as percentages relative to untreated cells. **d** Experiments similar to (**c**) performed in PERK KO MEL526 cells. For all panels, shown is a representative experiment of four independent repetitions. Quantification is the average of four independent experiments ± SE

In the original document that described GSK414 as a specific inhibitor for PERK, KIT was listed at the top of its potential targets, right after PERK, with an estimated IC_50_ of 154 nM obtained in a cell-free assay. PERK was inhibited by GSK414 at sub-nanomolar concentrations, indicating selectivity of at least two orders of magnitude between the on-target and off-target inhibition^5^. However, multiple studies in living cells using GSK414 indicated that complete inhibition of PERK by GSK414 occurred at concentrations ranging from 250 nM to 1 µM^14–16^. At this concentration range, KIT in living cells may be affected. First, we measured the direct binding of GSK414 to KIT in vitro. As a positive control we used imatinib, a clinically used KIT inhibitor^17^. KIRA6 is structurally similar to GSK414 (Fig. 1a) and was used intentionally as an analog that is not expected to bind KIT. In the direct binding assay, based on MST, the calculated *K*_*d*_ of imatinib was 383 ± 146 nM, while GSK414 *K*_*d*_ was 664 ± 294 nM. As expected, KIRA6 had a *K*_*d*_ value of 10.8 ± 2.9 µM (Fig. 1b). The value for imatinib was consistent with its published IC_50_ for KIT^18,19^, providing credibility for this method. We then assayed the phosphorylation of KIT in a melanoma cell line (MEL526) that endogenously expresses KIT upon stimulation with SCF. In the presence of 250 nM of GSK414, P-KIT dramatically decreased as assessed by Western blotting with anti-PY-KIT antibodies. This effect was comparable to 40 nM of dasatinib estimated to inhibit wt KIT at an IC_50_ of 10–20 nM (Fig. 2a)^20,21^. GSK414 inhibited KIT in living cells at nM concentrations, comparable to the *K*_*d*_ values from the in vitro binding assay. Thus, caution should be taken in ascribing the biological effects of GSK414 to PERK only at sub-micromolar concentrations.

We then escalated GSK414 concentrations and time of treatment. Following overnight treatment, we observed a dose-dependent reduction in the total levels of KIT, which was accompanied by a dose-dependent reduction of cell surface KIT. Dasatinib, on the other hand, did not affect KIT expression, suggesting that inhibition per se of KIT is not sufficient for its downregulation (Fig. 2b, c). The fact that the downregulation of KIT expression plateaued at approximately 4 µM of GSK414 (Fig. 2c), suggested that this effect is most likely not dependent on PERK. This was indeed confirmed in PERK KO cells which display a similar plateau concentration (Fig. 2d).

GSK414 is not the only PERK inhibitor. Additional inhibitors with similar affinities have been developed by Amgen and Eli Lilly^22,23^. To examine if KIT inhibition is shared by other PERK inhibitors we analyzed Amgen’s molecule AMG PERK 44 (labeled as AMG44). Up to 20 µM of concentration, AMG44 did not block KIT phosphorylation following SCF stimulation and did not reduce KIT cell surface expression. In fact, we even observed a modest increase in KIT expression upon treatment with AMG44 (Fig. S1). Recently, GSK414 was shown to inhibit RIPK1^7^. To exclude that the effect we observed for KIT is not related to this off-target activity, we used the well-defined RIPK1 inhibitor necrostatin 1. Concentrations up to 100 µM neither affected KIT surface expression nor its phosphorylation (Fig. S1). Taken together, we conclude that inhibition of KIT is an off-target effect of GSK414 and that the interaction with the drug leads to the downregulation of the receptor, an unusual feature for an antagonist.

### GSK414 promotes the endocytosis of KIT and directs it for lysosomal destruction

**Fig. 3:**
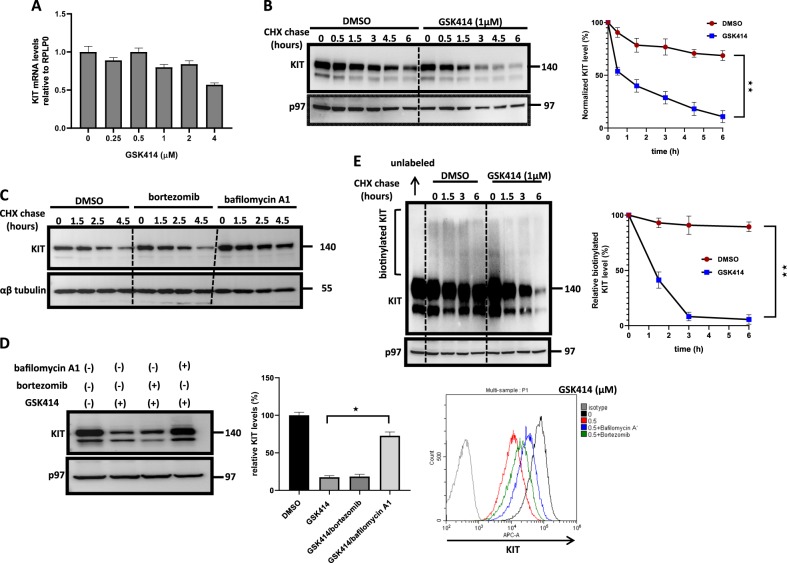
GSK414 specifically promotes the lysosomal degradation of surface KIT. **a** qPCR analysis for KIT mRNA levels relative to RPLP0 as housekeeping gene for MEL526 PERK KO following GSK414 treatments for 12 h. Shown is the average ± SD of three independent experiments. No significant change in the mRNA level of KIT when cells treated by GSK414 was observed. **b** Immunoblotting for KIT following cycloheximide chase of MEL526 PERK KO pretreated for 1 h either with DMSO or with GSK414. Quantification of total KIT levels relative to p97 as loading control from four independent experiments, expressed as percentage from the zero time point. Significance at *p* < 0.01 is shown. **c** Immunoblotting for KIT following cycloheximide chase of MEL526 PERK KO pretreated for 2 h with DMSO, Bortezomib (300 nM), or bafilomycin A1 (150 nM). **d** Immunoblotting and flow cytometry analysis for surface KIT for GSK414 (0.5 µM) treated cells for 16 h followed by the addition of DMSO, bortezomib, or bafilomycin A1 during the last 2 h of the experiment. Quantification of three independent experiments is shown (*p* < 0.05). **e** Immunoblotting for KIT under non-reducing conditions showing surface biotinylated KIT as a smear in MEL526 PERK KO followed by 6 h cycloheximide chase in the presence of DMSO or GSK414 (1 μM). Quantification of three independent repetitions is shown ± SE (*p* < 0.01)

The effects of GSK414 on KIT stability may be not restricted to KIT. We examined the effect of GSK414 on the surface level of other glycoproteins by flow cytometry. Surface expression of three other RTKs, IGFR, C-MET, and EGFR, was not affected by GSK414. Moreover, the dynamics of transferrin receptor, which is subjected to endosomal recycling, remained unaltered (Fig. S2). This indicates that the effect of GSK414 on KIT is not shared by other cell surface RTKs and glycoproteins. Because GSK414 did not affect the mRNA levels of KIT to the extent that it affected the protein level (Fig. 3a), we hypothesized that GSK414 impinges on KIT stability as its mechanism of downregulation. In DMEM media supplemented by 10% FBS, KIT is not stable and its level decreases upon cycloheximide addition over time. However, the degradation was strongly enhanced in the presence of GSK414 (Fig. 3b). The inclusion of the proteasome inhibitor, bortezomib, to the chase had no effect on KIT degradation in the presence of GSK414. In contrast, the Na/H exchanger inhibitor of endosome acidification bafilomycin A1 completely stabilized KIT (Fig. 3c). This suggests that GSK414 accelerates the lysosomal destruction of KIT, a conclusion supported by the significant elevation of KIT levels and restoration of surface KIT when bafilomycin A1 was added to GSK414 treatment (Fig. 3d). This was further supported by following the degradation of surface KIT. Gentle surface biotinylation showed a high molecular weight smear of KIT proteins. The disappearance of the smear (biotinylated KIT) in the course of the cycloheximide treatment was accelerated in the presence of GSK414 in accordance with total KIT levels (Fig. 3e). We also confirmed that the ER-localized KIT is not affected by GSK414 by assessing the stability of the D816V mutant of KIT, which is arrested in the ER in 293T cells. The expression level of the D816V mutant was not affected by GSK414 while wild-type KIT levels were reduced by GSK414 in a dose-dependent manner (Fig. S3). Microscopy images for KIT confirmed intracellular accumulation of the protein in the presence of GSK414 without affecting the total lysosomal content, as accounted for by LysoTracker staining (Fig. S3). Together, our data indicate that GSK414 selectively and efficiently promotes KIT endocytosis and lysosomal clearance while being an antagonist.

### KIRA6 inhibits KIT without affecting its expression levels and protects KIT from GSK414-mediated degradation

**Fig. 4:**
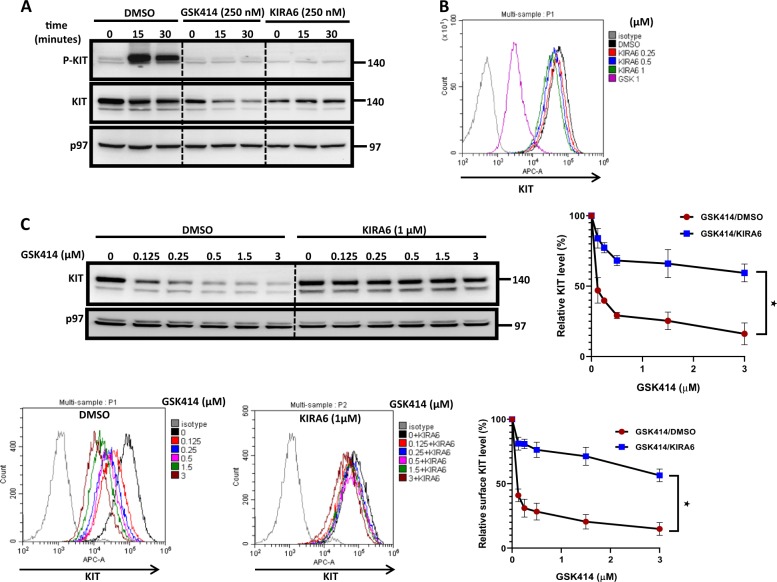
KIRA6 inhibits KIT without affecting its expression levels, exhibiting antagonism properties for GSK414-promoted KIT degradation. **a** Time course immunoblotting for P-KIT and total KIT in MEL526 PERK KO cells, pretreated for 1 h with DMSO, GSK414, or KIRA6 followed by SCF addition (50 ng/ml) for up to 30 min. **b** Flow cytometry analysis for surface KIT in MEL526 PERK KO cells following increasing concentrations of KIRA6 or GSK414 for 10 h. **c** Immunobloting (top) and flow cytometry analysis (bottom) for KIT in PERK KO cells, treated with increasing concentrations with GSK414 alone or in combination with KIRA6 (1 μM) for 12 h. KIT flow cytometry quantifications are expressed as percentage from untreated cells. Quantification is the average of three independent experiments ± SE

Though postulated not to affect KIT signaling at sub-micromolar concentrations, we noticed that KIRA6 also blocks KIT phosphorylation at concentrations similar to that of GSK414 (Fig. 4a). However, in contrast to GSK414, KIRA6 did not affect surface KIT levels (Fig. 4b). Moreover, GSK414 in the presence of KIRA6 (1 μM) hardly affected the expression level of KIT (Fig. 4c). This effect was independent of IRE1, as similar results were obtained in IRE1 KO cells (Fig. S4). These data show that KIT is a shared target for two UPR pharmacological inhibitors in an off-target manner and further highlight the unusual downregulation effect of GSK414 on KIT.

### Both GSK414 and KIRA6 display different interaction in the active site of KIT

**Fig. 5:**
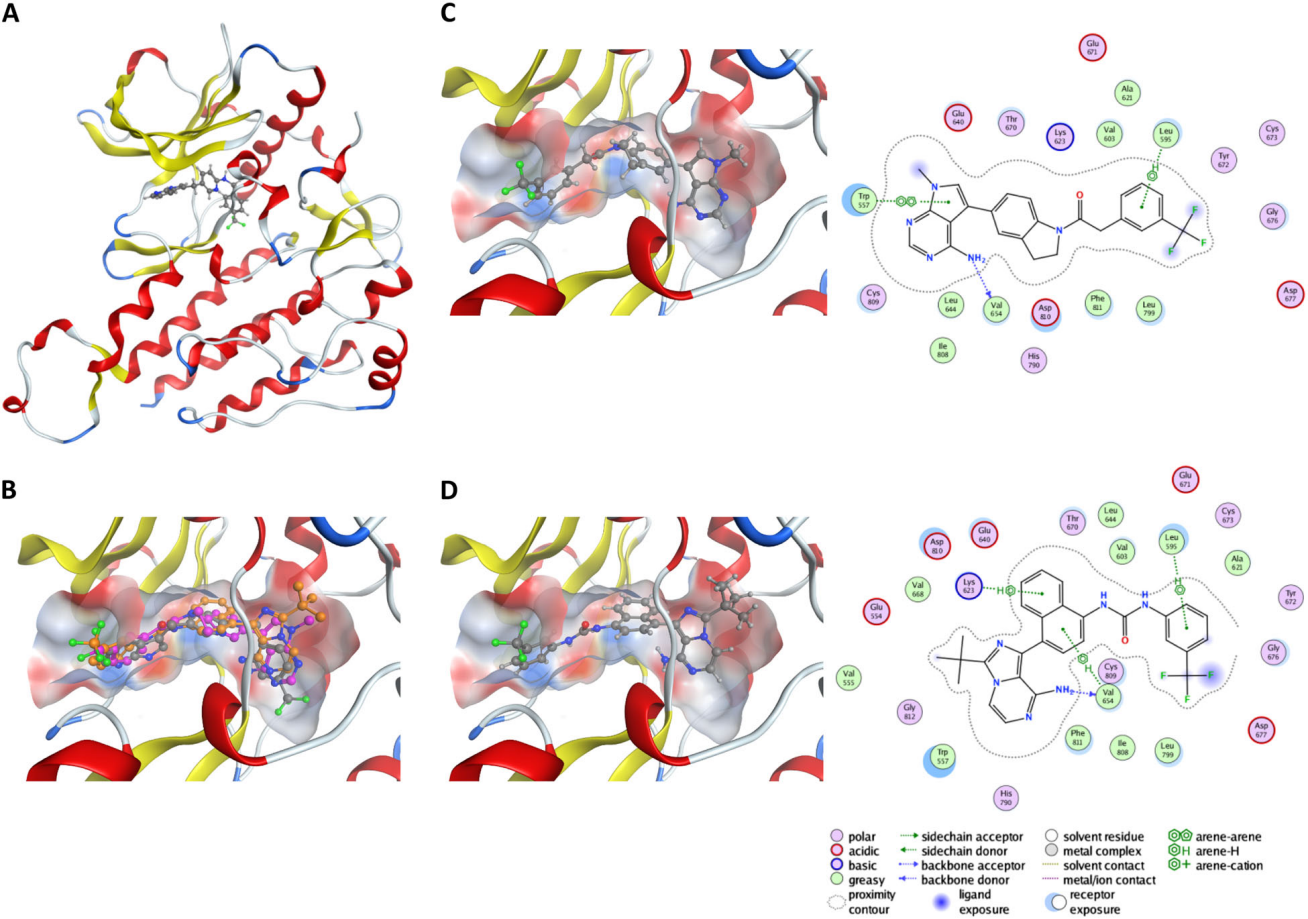
GSK414 and KIRA6 dock with high stringency to the active site of KIT, but display different interaction patterns. **a** KIT co-crystallized with PLX647 (PDB-ID 4HVS). **b** Active site of KIT, with the best docked structures of PLX647 (carbon atoms colored gray), GSK414 (carbons colored magenta), and KIRA6 (carbons colored orange). **c** The best docked structure of GSK414 and its 2D interaction map. **d** The best docked structure of KIRA6 and its 2D interaction map, including legends

To further address the interaction of GSK414 and KIRA6 with KIT and provide atomistic level insight for the distinct effect of the drugs on KIT stability, we performed in silico docking of these two drugs on the crystal structure of KIT in complex with PLX647, an experimental KIT inhibitor^13^. Several crystal structures of KIT exist, and given the plasticity of the active site, the structure co-crystallized with the ligand bearing closest resemblance to GSK414 and KIRA6, PDB-ID 4HVS, was chosen. When docked into the active site of KIT, GSK414 and KIRA6 undertake similar orientations (Fig. 5), yet with different interaction patterns to the residues lining the active site. They both adopt a “reverse” orientation relative to ADP/ATP and to PLX647, in that the purine-like fused ring is placed at the phosphate binding region, and the trifluoro methyl benzene moiety in the adenine binding part of the active site. Both molecules display hydrogen bonded interaction from the amine group of the fused ring to the backbone of Val654, and hydrophobic arene–hydrogen interaction between the aromatic ring of the trifluoro methyl benzene moiety and the side chain of Leu595. KIRA6 also has arene–hydrogen interaction between the central naphthalene unit with Val654 and the side chain of Lys623, whereas for GSK414 the terminal fused ring system forms π-stacking interaction with Trp557. For KIRA6, the bulky isobutyl substituent hinders this interaction and displace the molecule somewhat relative to GSK414. Notably, both compounds show very similar docking scores, supporting the finding that they are able to compete for binding to the active site.

### Both GSK414 and KIRA6 compromise the viability of KIT-dependent HMC-1.1 cells

**Fig. 6:**
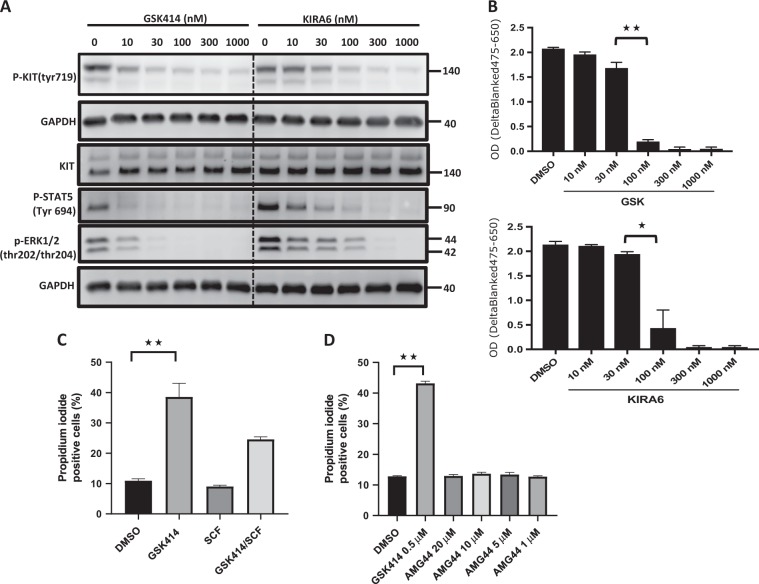
GSK414 blocks KIT signaling and exhibits toxicity to HMC-1.1. **a** Immunoblotting of KIT signaling in HMC-1.1 cells treated with increasing concentrations of GSK414 and KRA6 for 1 h. **b** XTT viability assay of HMC-1.1 treated with different concentrations of GSK414 or KIRA6 for 72 h. Shown is the average ± SD of three independent experiments (***p < 0.01, **p < 0.05). **c** The percentage of propidium iodide (PI) positive cells, as measured by flow cytometry following HMC-1.1 treatment with DMSO, GSK414, and SCF (50 ng/ml) alone or in combination with GSK414 for 12 h. Shown is the average ± SD of three independent experiments (*p* < 0.01). **d** The percentage of propidium iodide (PI) positive cells, as measured by flow cytometry following HMC-1.1 treatment with DMSO, GSK414, and escalating concentrations of AMG44. Shown is the average ± SD of three independent experiments (*p* < 0.01)

KIT is a major pro-survival receptor for certain types of cells, and thus its inhibitors are used in cancer therapy. Because the pharmacological evidences of GSK414 and KIRA6 were obtained in melanoma cells that do not rely on KIT signaling for survival, we tested the pharmacological usefulness of GSK414 and KIRA6 as KIT inhibitors for therapy. This was carried out using mast cell leukemia cell line HMC-1.1, which relies on KIT signaling for survival and is highly sensitive to various KIT inhibitors, such as imatinib, dasatinib, and nilotinib^24,25^. Short treatment of HMC-1.1 with concentrations ranging from 10 nM to 1 µM of the drugs showed that already at 100 nM, signaling output of KIT was reduced, including the phosphorylation of KIT as well as its downstream signaling modules, P-STAT5 and phosphorylated ERK1/2 (Fig. 6a). This was accompanied by reduced cellular viability assessed by XTT assay after 72 h (Fig. 6b) and propidium iodide staining (not shown). Cell death was partially rescued by the addition of SCF (Fig. 6c). Because the inhibitors in this cell line were also inducing cell death, it was not possible to clearly determine the effect of the treatments on the surface expression levels of KIT. For dasatinib, with similar data obtained for GSK414 and KIRA6 (not shown), within 8 h of treatment, we recorded reduced levels of surface KIT which segregated primarily to the dead cells (Fig. S5). As expected AMG44, which does not block KIT, did not compromise HMC1.1 viability even at a concentration of 20 µM (Figs. 6d and S6). We conclude that in living cells, GSK414 and KIRA6 are potent KIT inhibitors at sub-micromolar concentration, and that the pharmacological activity in most cell types cannot be distinguished from PERK or IRE1 inhibition, respectively.

## Discussion

The development of specific kinase inhibitors is a formidable task, as most, if not all, clinically approved drugs bind to the ATP binding site of the targeted kinase, which often resembles between enzymes in an unpredicted fashion. While clinically the ability to target simultaneously a few kinases may offer therapeutic advantages, particularly for cancer treatment, for research purposes this confounds mechanistic understanding. In many cases, to assess the specificity of the kinase inhibitor, a cell-free assay is employed, which provides a measure of the binding constant to the protein in question. This method is regarded free from biases such as cell identity, membrane permeability, off-target binding, biotransformation and dynamics of expression, which affects the IC_50_ calculations^26^. For most molecules, the effective concentrations in living cells are higher than for the purified protein. However, a major caveat of this approach is the use of only the soluble cytosolic portion of RTKs, disconnected from the orientation of receptors in the cellular membrane and the loss of cellular regulators. Pertain to this study, KIT activity is regulated by multiple phosphorylations and by a constitutive interaction with SHP-1^27^, all are not accounted for in the in vitro binding assays (Fig. 1). This, and probably other factors, such as the ability of TKIs to accumulate intracellularly and acquire a higher local concentration than was added^28^, may explain the block of KIT at sub-micromolar concentrations by GSK414 (Fig. 2) and KIRA6 (Fig. 4). It should be emphasized that most studies of UPR utilize these inhibitors in this pharmacological concentration range.

The expression levels of many receptors are sensitive to their triggering. G-protein coupled receptors undergo rapid endocytosis following agonist stimulation, a response so strong, that it is utilized clinically to achieve loss of function by agonists, such in the case of GnRH receptor^29^. For some RTKs, such as EGFR, the mechanisms of endocytosis and delivery to the lysosomes following ligand stimulation have been thoroughly studied^30^. The initial signal for endocytosis is dimerization of the receptor, which instigates the recruitment of ubiquitin E3 ligases and the ubiquitination of the receptor. The levels and density of the ubiquitinated receptor determine whether it will be recycled back to the cell surface, or directed to late endosome en route to the lysosome. While most RTKs signal from the cell surface, others, like C-MET, requires endocytosis for their signaling and a correlation between endocytosis and output signal has been demonstrated^31,32^. Surface expression levels of KIT is highly dynamic. In the presence of SCF, KIT is endocytosed into early endosomes and then directed to late endosomes. This trafficking is dependent on clathrin assembly^33^. Attempts to visualize KIT by appending GFP to its C-terminus showed that this modification was sufficient to activate KIT and promote its degradation in a ligand-independent fashion^34^. Our data confirmed the reduction in KIT expression following SCF stimulation (Fig. 2). However, examples in which RTKs antagonists promote degradation are rare. Thus, the downregulation of KIT by GSK414 is unusual (Figs. 2 and 3). However, analysis of surface KIT levels in hematopoietic cells treated with the inhibitors dasatinib or radotinib showed the downregulation of KIT. It should be noted that this reduction coincided with severe cytotoxicity, confounding the interpretation of the experiment^35^. We also observed that in HMC-1.1 cells, which die upon KIT inhibition, surface levels of KIT were reduced upon treatment with all tested inhibitors (Fig. S5). However, in cells that do not rely on KIT activity for survival, dasatinib did not affect the surface level of KIT (Fig. 2). We conclude that in all cases KIT downregulation is instigated by agonistic stimulation and not by its clinically used inhibitors. However, in contrast to dasatinib, GSK414 unequivocally caused the downregulation of KIT in the melanoma cells. Moreover, KIRA6, while being a potent inhibitor of KIT in vivo, did not affect its expression level as dasatinib, and even negated GSK414-mediated downregulation of KIT (Fig. 4). Because the degradation of KIT occurred only for the surface located molecules (Fig. 3), our data indicate that at least for KIT, endocytosis can be facilitated using an inhibitor. A plausible reason is that GSK414 stabilizes a conformation that is recognized by the cell as resembling an active KIT, while KIRA6 and dasatinib do not. Analysis of the orientation of GSK414 and KIRA6 in the active site of KIT by molecular modeling indicated that while both GSK414 and KIRA6 are enwrapped tightly in the ATP-binding pocket, the isobutyl substituent of KIRA6 displaces the molecule somewhat, leading to differences in interactions to the active site residues that could possibly mediate the different intracellular response observed. Both molecules strongly compromised the viability of the KIT-dependent cell line HMC-1.1 at the low nM concentration, in a manner that coincided with KIT blockade (Fig. 6), which is consistent with their similar docking scores.

As mentioned above, the mostly used PERK inhibitor, GSK414, is apparently not specific and some of the side effects attributed to this drug in vivo, can be a result of its off-target activities, rather than the targeting of PERK. From our preliminary analysis, at least with respect to KIT inhibition, the PERK inhibitor AMG44 is apparently more specific (Fig. S1), and thus may be better suited for clinical applications. The fact that KIRA6 inhibited KIT efficiently, was unexpected and pertains directly to a recent study, which implicates the cross-interaction of IRE1 and cytosolic ABL tyrosine kinases^36^. This study demonstrated that inhibition of ABL by imatinib, also a potent KIT inhibitor, affected IRE1 kinase activity, and vice versa, the kinase activity of IRE1 alleviated ABL mediated UPR activation^36^. If the IRE1 and ABL inhibitors cross-react in vivo, as inferred from our study, this cross-talk should be reexamined.

## Supplementary information

Figures S1-S6
