# Supplementary material for: The unfolded protein response modulators GSK2606414 and KIRA6 are potent KIT inhibitors
Source: Cell Death Dis. 2019 Apr 1;10(4):300. doi: 10.1038/s41419-019-1523-3 (PMC6443726; doi:10.1038/s41419-019-1523-3)
Supplement: Supplementary file 1 — Figures S1-S6 [file 41419_2019_1523_MOESM1_ESM.pdf]

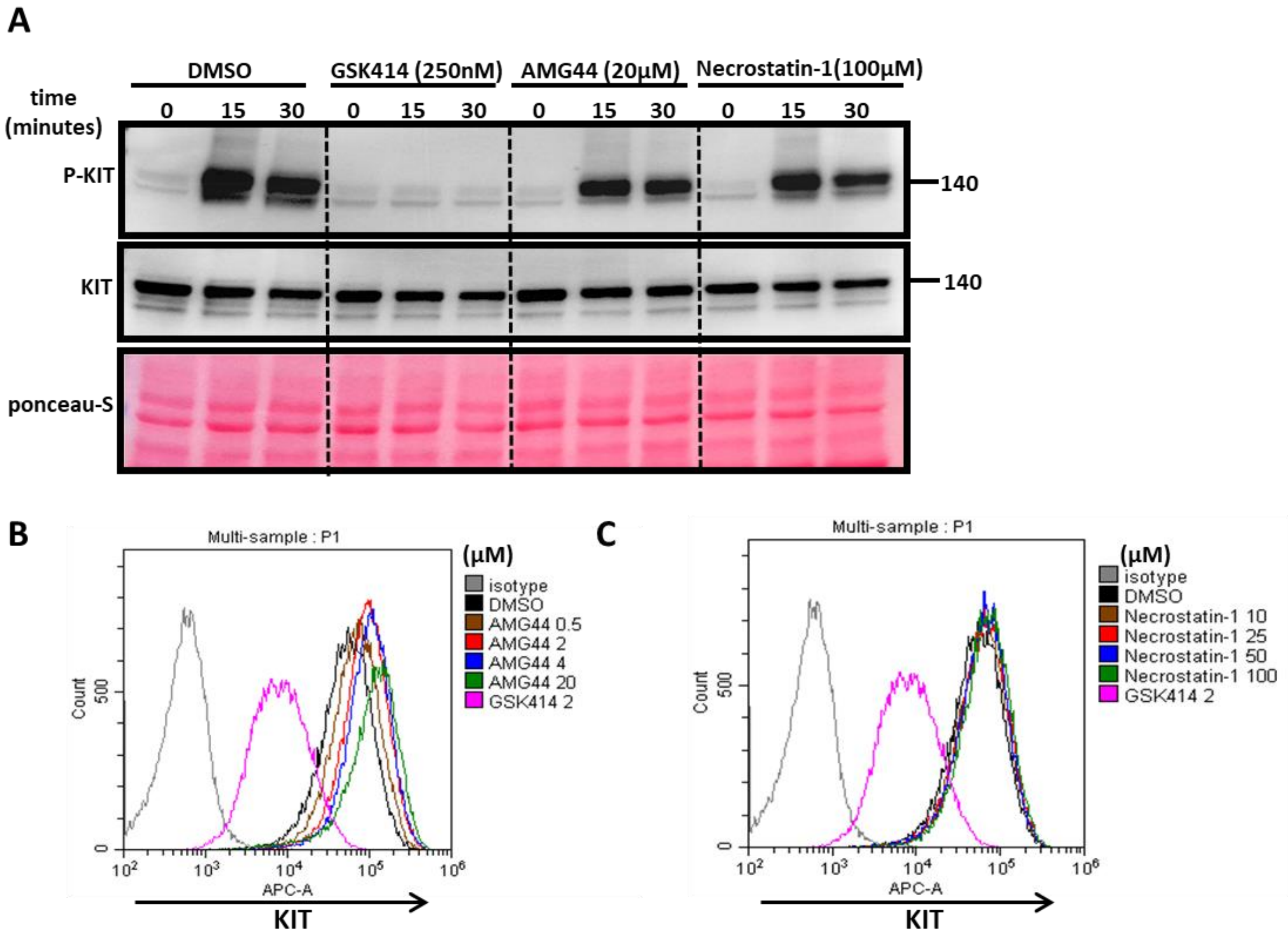

Figure S1: **AMG44 and necrostatin-1 do not block KIT. (A)** Immunoblotting of MEL526 treated with SCF (50 ng/ml) in the presence of GSK414, AMG44 and Necrostatin-1, added 1h before and during the SCF stimulation. (**B and C**) Flow cytometry analysis of MEL526 surface KIT levels following AMG44 or necrostatin-1 treatments for 16h. GSK414 2µM was added as positive control for both experiments.

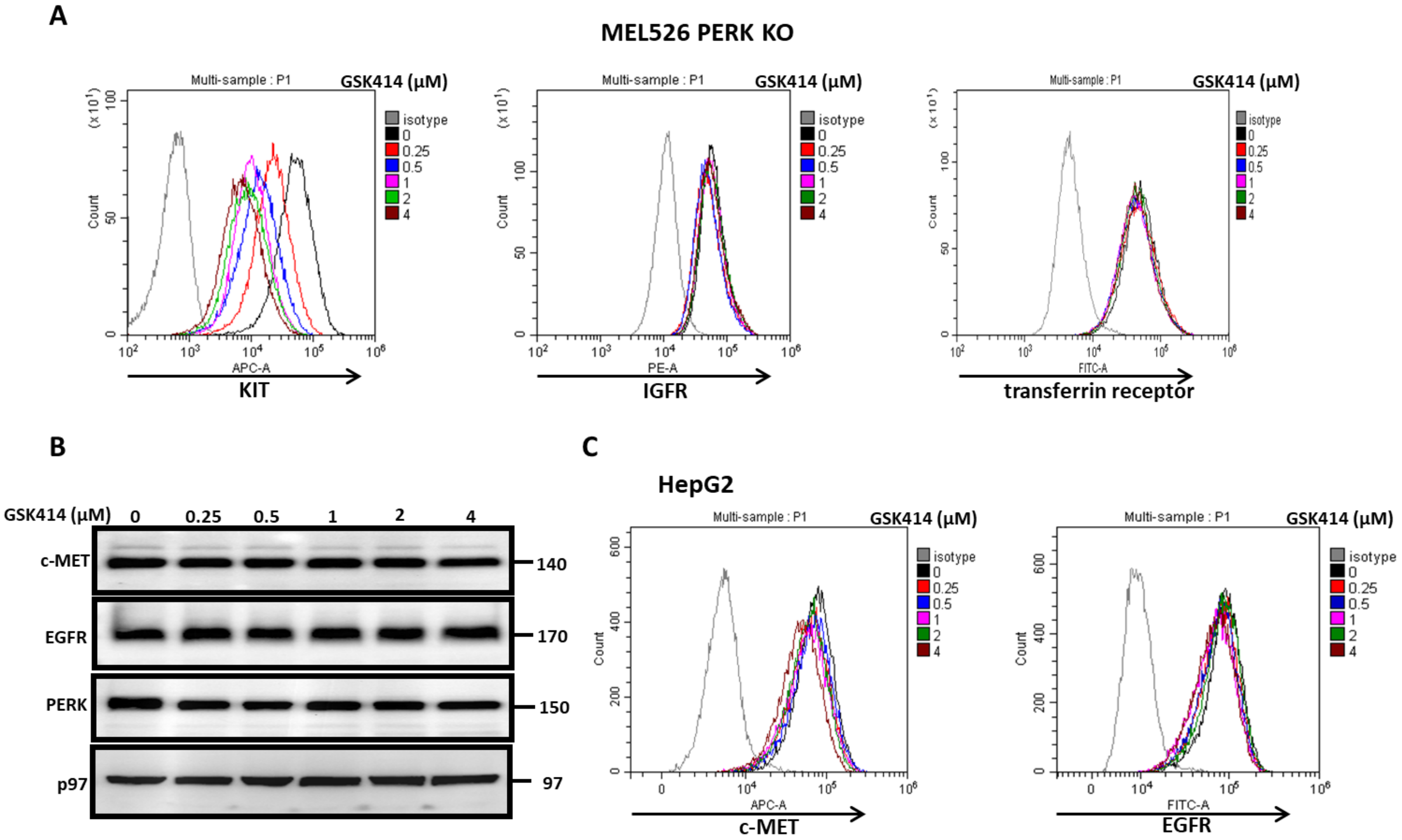

Figure S2: **GSK414 selectively downregulates KIT.** **(A)** Flow cytometry analysis of KIT, IGFR, and transferrin receptor in MEL526 PERK KO following GSK414 treatments for 16h. **(B)** Immunoblotting and flow cytometry **(C)** for c-MET and EGFR in hepG2 cells treated with different concentrations of GSK414 for 16h.

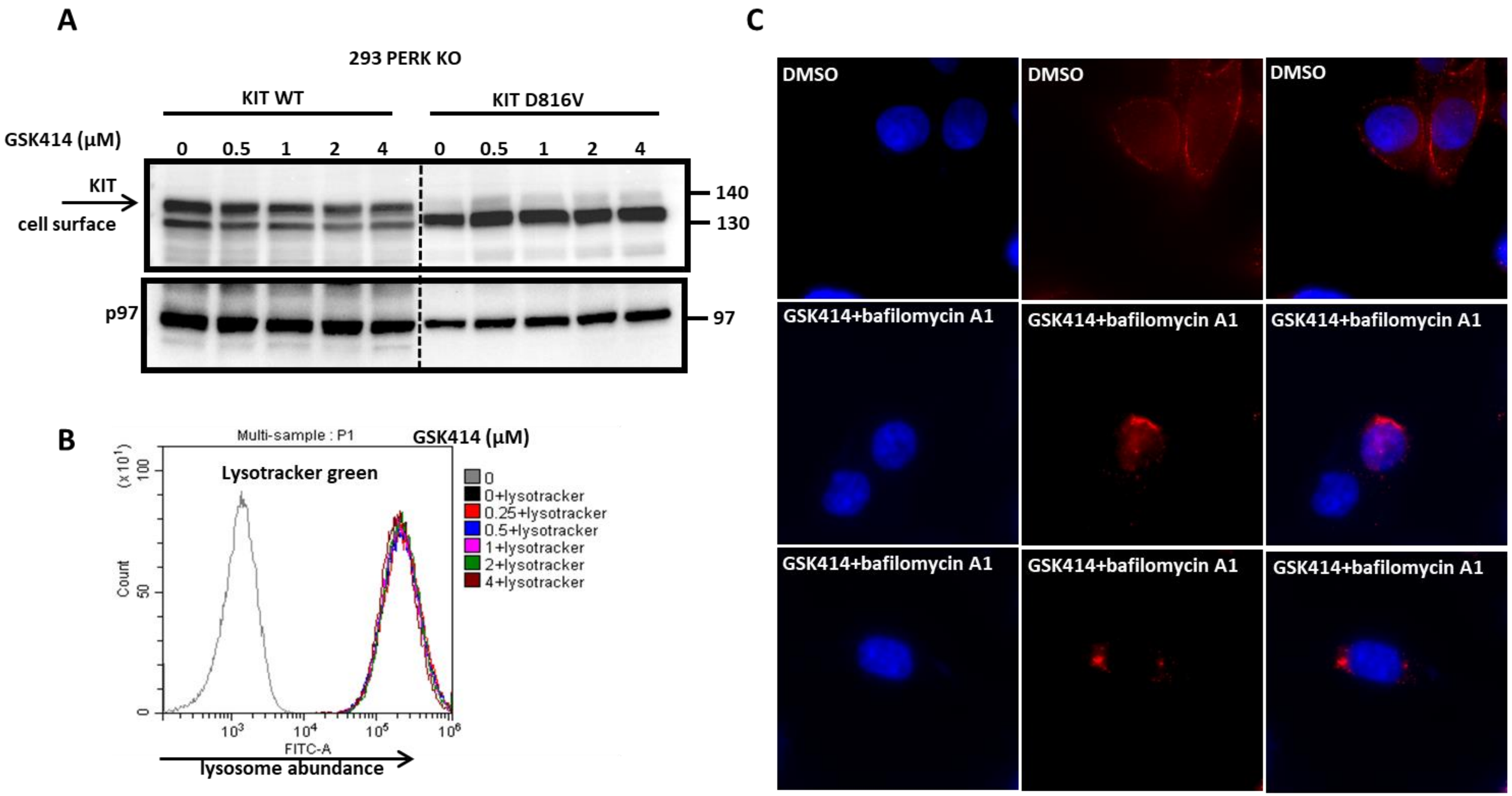

Figure S3: **GSK414 enhances the lysosomal degradation of KIT. (A)** KIT immunoblotting of 293T cells transfected either with KIT WT or with KIT D816V mutant followed by 16h treatment with increasing concentrations of GSK414 **(B)** Flow cytometry analysis for MEL526 cells stained with lysotracker green following GSK414 treatments for 16h. **(C)** KIT immunofluoresence (in red) for MEL526 treated either with DMSO or GSK414 (1μM) accompanied with bafilomycin A1 (100 nM) for 12h. DAPI is shown in blue.

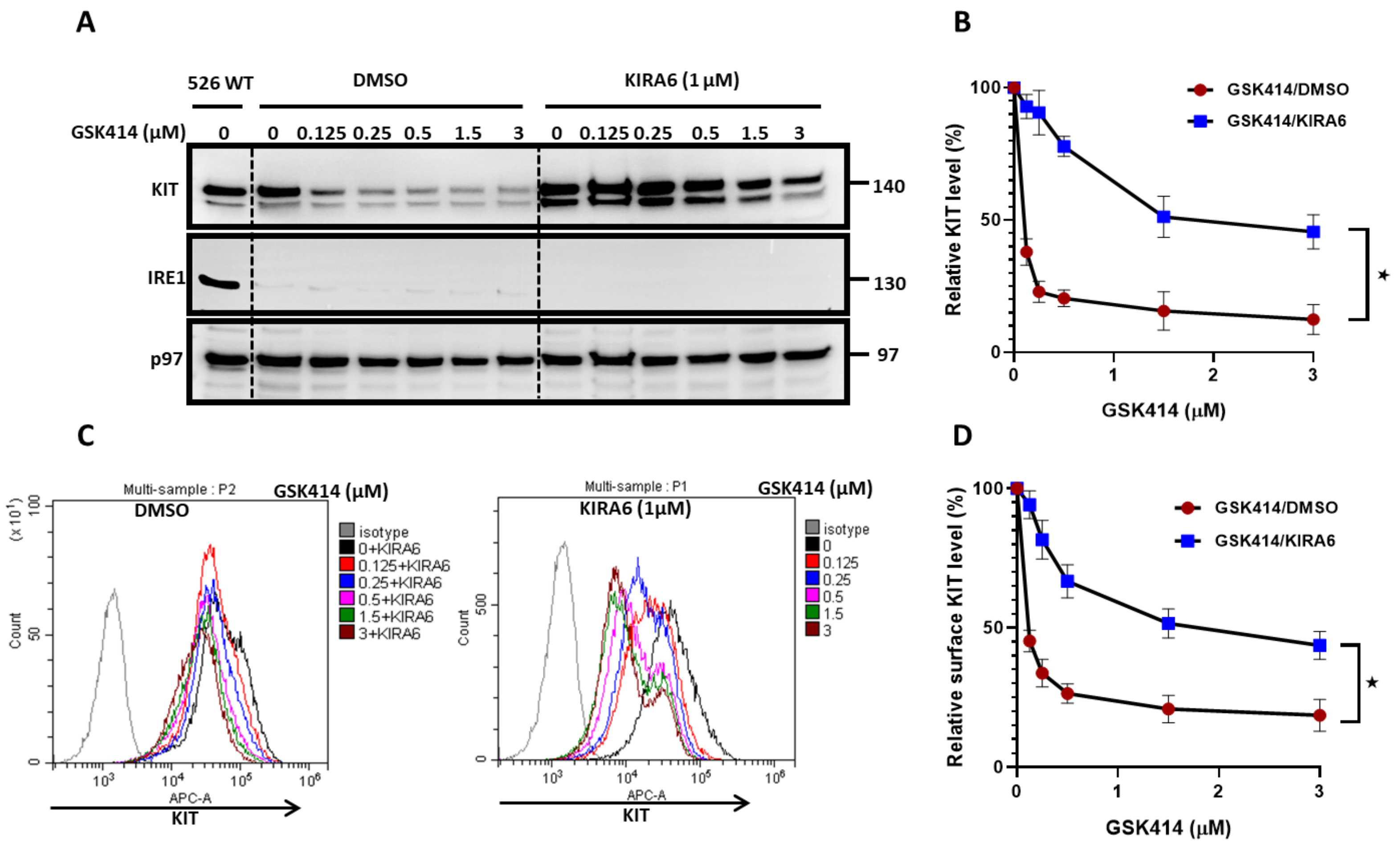

Figure S4: **KIRA6 inhibits GSK414-mediated KIT degradation independently of IRE1. (A)** KIT immunoblotting of IRE1 KO MEL526 upon GSK414 treatments in presence of DMSO or KIRA6 (1μM) for 16h **(B)** Quantification of KIT levels relative to p97 as loading control. Shown is the average of three independent experiments (p<0.05) **(C)** Flow cytometry analysis of the surface KIT levels from the previous experiment **(D)** Surface KIT levels based on mean fluorescence quantification, shown as KIT percentage (%) relative to vehicle treated cells. Shown is the average of three independent experiments (p<0.05).

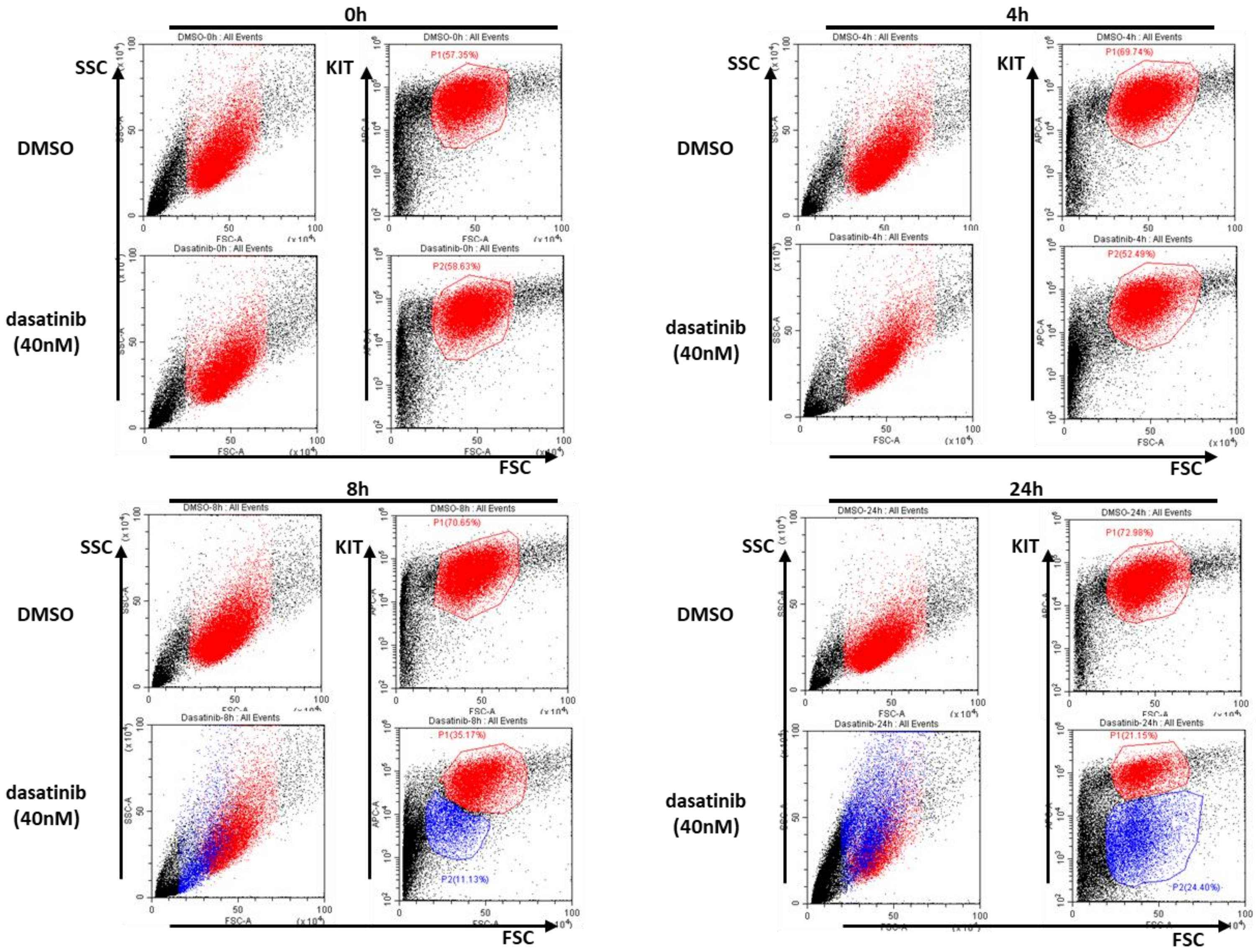

Figure S5: **Downregulation of KIT levels is associated with cell death in HMC1.1. (A)** Flow cytometry analysis of HMC1.1 treated either with DMSO or dasatinib (40 nM). For each time point, SSC versus FSC (left) and KIT levels versus FSC (right) are shown for both treatments. Shown is a representative experiment of three repetitions. Live cells are labeled in red. Dead cells are labeled in blue. Note the reduction in KIT expression following death.

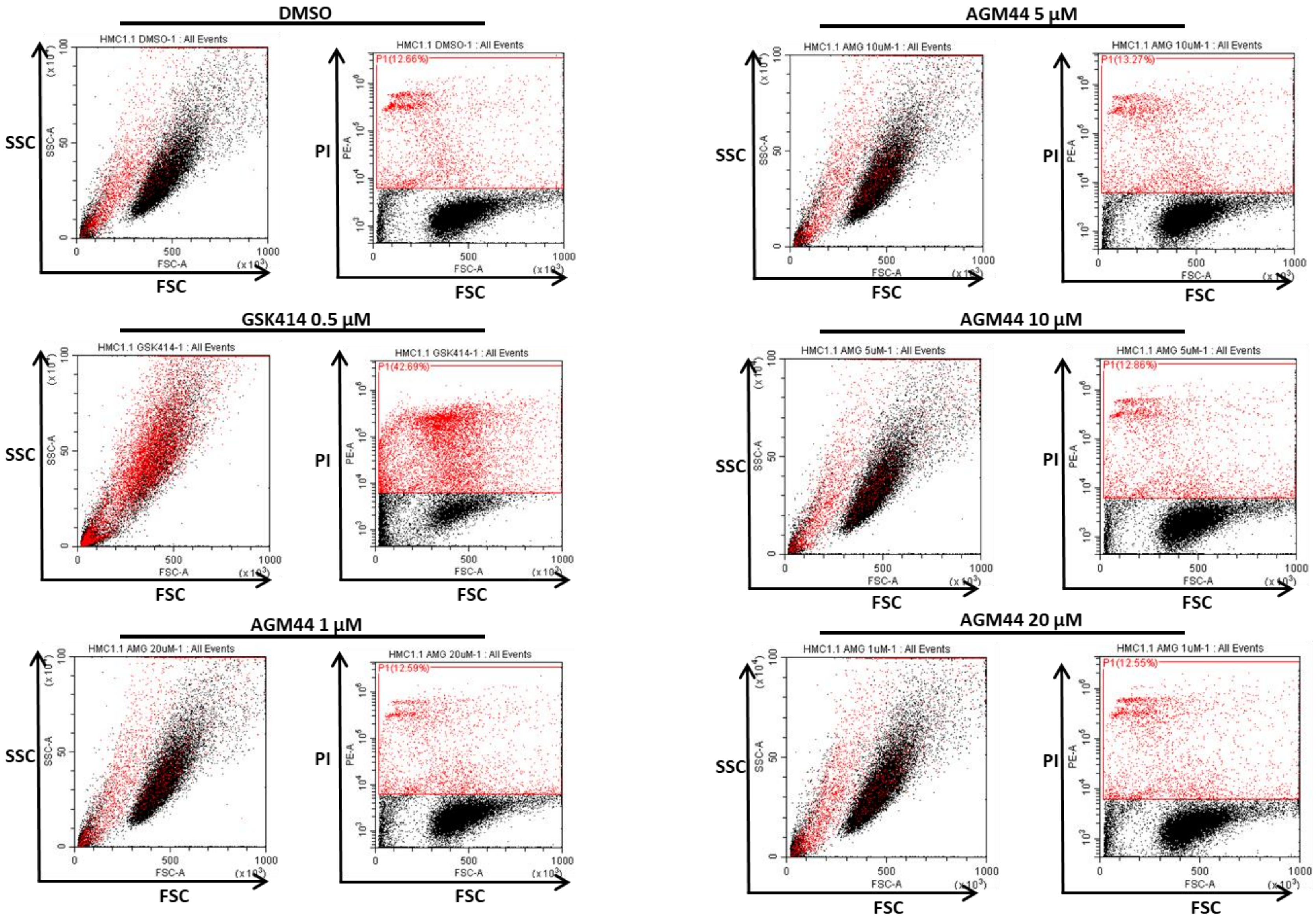

Figure S6: **AMG44 does not compromise HMC1.1 viability.** Flow cytometry analysis displayed as SSC vs. FSC (left) and PI staining (right) of HMC1.1 treated either with DMSO, GSK414 (0.5 μM), or with different concentrations of AMG44 for 18 hours. PI positive (dead) cells are displayed in red color. Shown is a representative experiment of three repetitions. 0.5μM of GSK414 served as a positive control.
